# Principles of Cell Circuits for Tissue Repair and Fibrosis

**DOI:** 10.1016/j.isci.2020.100841

**Published:** 2020-01-16

**Authors:** Miri Adler, Avi Mayo, Xu Zhou, Ruth A. Franklin, Matthew L. Meizlish, Ruslan Medzhitov, Stefan M. Kallenberger, Uri Alon

**Affiliations:** 1 Department Molecular Cell Biology, Weizmann Institute of Science, Rehovot 76100, Israel; 2 Broad Institute of Massachusetts Institute of Technology and Harvard, Cambridge, MA 02142, USA; 3 Howard Hughes Medical Institute Department of Immunobiology, Yale University School of Medicine, New Haven, CT 06510, USA; 4 Digital Health Center, Berlin Institute of Health (BIH) and Charité, Berlin 10178, Germany; 5 Division of Theoretical Bioinformatics, German Cancer Research Center (DKFZ), Heidelberg 69120, Germany

**Keywords:** In Silico Biology, Systems Biology, Tissue Engineering

## Abstract

Tissue repair is a protective response after injury, but repetitive or prolonged injury can lead to fibrosis, a pathological state of excessive scarring. To pinpoint the dynamic mechanisms underlying fibrosis, it is important to understand the principles of the cell circuits that carry out tissue repair. In this study, we establish a cell-circuit framework for the myofibroblast-macrophage circuit in wound healing, including the accumulation of scar-forming extracellular matrix. We find that fibrosis results from multistability between three outcomes, which we term “hot fibrosis” characterized by many macrophages, “cold fibrosis” lacking macrophages, and normal wound healing. This framework clarifies several unexplained phenomena including the paradoxical effect of macrophage depletion, the limited time-window in which removing inflammation leads to healing, and why scar maturation takes months. We define key parameters that control the transition from healing to fibrosis, which may serve as potential targets for therapeutic reduction of fibrosis.

## Highlights

• A myofibroblast-macrophage circuit for tissue repair shows three physiological states
• States correspond to healing, cold fibrosis (lacking macrophages), and hot fibrosis
• The circuit explains how a transient/persistent insult leads to healing/fibrosis
• The PDGF autocrine loop is a potential target for abrogating fibrosis

## Graphical Abstract

**Figure:**
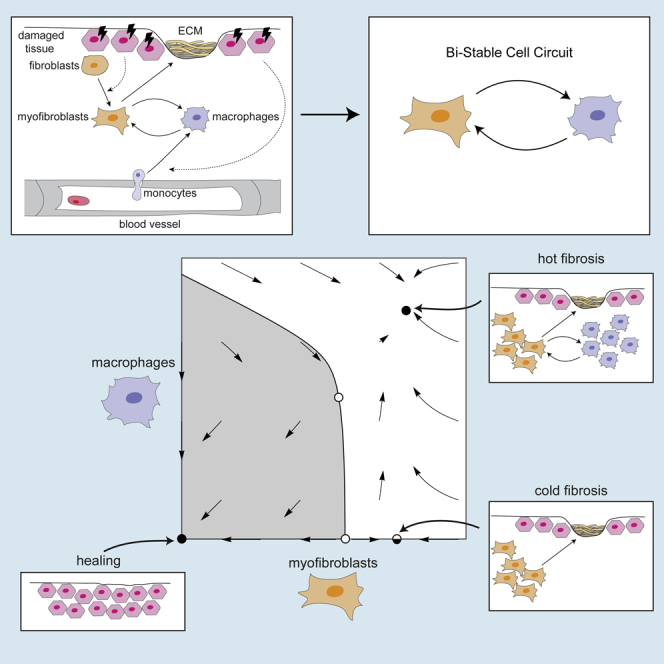

## Introduction

**Figure 1:**
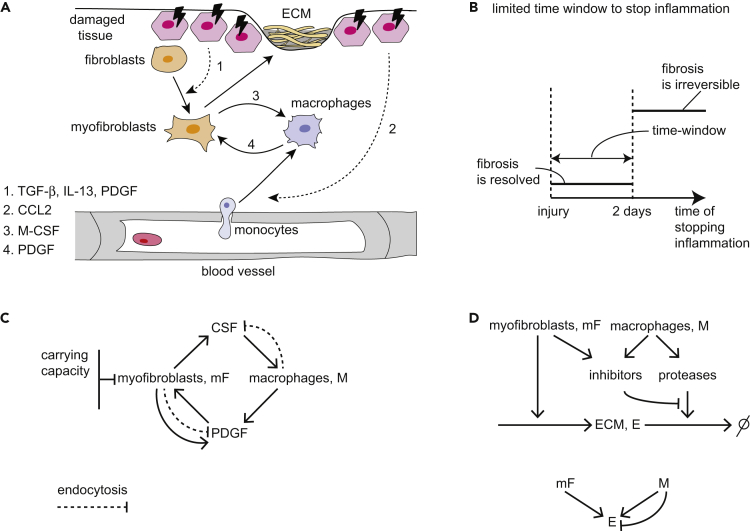
Overview of Cell-Cell Interactions Following Tissue Injury (A) Schematic of wound healing and scar formation. (B) If injury is transient, it leads to brief inflammation, and no fibrosis occurs. However, if injury is persistent and inflammation exceeds a critical time window, fibrosis is usually inevitable. (C) Circuit in which myofibroblasts (mF) and macrophages (M) secrete growth factors (GFs) for each other; mFs show an autocrine loop and are limited by a carrying capacity. Both cell types remove the GFs by endocytosis (dashed arrows). (D) ECM is produced by mF and degraded by proteases mainly secreted by M. Proteases are inhibited by factors secreted by mF and M.

Tissue injury initiates a dynamic process that involves an immune response, local proliferation of cells, scar deposition, and tissue regeneration. Following an injury, signals from the damaged cells recruit inflammatory cells, such as monocytes and neutrophils, and stimulate differentiation of fibroblasts into myofibroblasts. Myofibroblasts produce extracellular matrix (ECM) proteins such as collagen and fibronectin that form the scar. Recruited monocytes, together with tissue-resident macrophages, differentiate into inflammatory macrophages that break down and engulf the fibrin clot and cellular debris (Figure 1A).

Injuries are classified according to the ability of the tissue to regenerate and the severity of the wound (Robbins, 1994). When the tissue has regenerative potential, such as many epithelial tissues, and the injury is mild, the inflammation is resolved within days, myofibroblasts and inflammatory macrophages are removed, and the scar gradually degrades over weeks. Injured epithelial cells are replaced through regeneration and normal tissue composition is restored (Diegelmann and Evans, 2004, Duffield et al., 2013, Gurtner et al., 2008, Mann et al., 2011, Mutsaers et al., 1997).

When the injury is more severe or persistent, however, regeneration is not possible and the organism resorts to repair with fibrosis. Scars mature over months into an aggregate of ECM, myofibroblasts, and macrophages. Fibrosis resolves the acute injury, but fibrotic tissue is less functional than the original tissue. Fibrotic diseases are a common cause of age-related decline in organ function and ultimately of organ failure. Fibrotic diseases appear in many tissues including the skin, the musculoskeletal system, lung, liver, heart, and kidney (Wynn, 2008).

Fibrosis often accompanies repetitive injury or chronic wounds with insufficient vascular supply. Prolonged injury can be due to persistent damaging agents, such as hepatitis C in the liver, or a failure to replace damaged cells, which prolongs the inflammatory response (Cao et al., 2016, Degryse et al., 2010, Mouratis and Aidinis, 2011). Such failure is especially common in tissues with poor regenerative capacity such as the heart.

In tissues with regenerative capacity, injury can thus lead to either regeneration or fibrosis, depending on the duration of the inflammatory signal. Studies show that there is a limited time-window of several days in which stopping inflammation avoids fibrosis (Figure 1B). Beyond this time-window, fibrosis inevitably occurs even if inflammation is stopped. For example, in viral infection, early removal of the inflammatory trigger by antiviral drugs improves the patient state and reduces fibrosis (Ferguson and O'Kane, 2004, Wynn and Ramalingam, 2012). Similarly, early macrophage depletion substantially reduces the development of liver fibrosis (Duffield et al., 2005, Ide et al., 2005, Pradere et al., 2013, Sunami et al., 2012). The origin of this time-window is unclear. In addition, the long timescale of scar maturation, which can take months, is surprising given this limited time-window, as well as the fact that cell turnover times are on the order of days.

In order to understand how a single process can lead to two very different physiological outcomes, healing and fibrosis, and to understand the timescales of fibrosis, it is important to define the circuit of cell-cell interactions that generates scarring. This circuit involves damaged tissue cells, myofibroblasts, and macrophages (Lech and Anders, 2013, Pakshir and Hinz, 2018, Ramachandran et al., 2019, Wynn and Barron, 2010, Wynn and Vannella, 2016). The macrophages can transition between different states, including pro-inflammatory M1 and anti-inflammatory M2 states (Braga et al., 2015, Sommerfeld et al., 2019).

In this circuit, macrophages and myofibroblasts reciprocally interact by growth factor exchange: myofibroblasts secrete colony-stimulating factor (CSF) for macrophages (CSF1, M-CSF) and macrophages secrete platelet-derived growth factor (PDGF) for myofibroblasts (Davies et al., 2013, Joshi et al., 2020, Lodyga et al., 2019, Wynn and Barron, 2010) (Figure 1A). In addition, myofibroblasts secrete PDGF in an autocrine loop that allows them to survive and expand in the absence of other growth-factor sources (Bonner, 2004, Trojanowska, 2008) (Figure 1C). This growth-factor communication is akin to the interaction between tissue-resident macrophages and fibroblasts that provides proper cell composition in the tissue, a circuit whose principles have been recently analyzed (Adler et al., 2018, Zhou et al., 2018).

Inflammatory macrophages also maintain the turnover of ECM by secreting factors that enhance or inhibit ECM degradation, namely matrix proteases (such as MMPs) and inhibitors of proteases (such as TIMPs). The latter are also secreted by myofibroblasts (Pellicoro et al., 2014) (Figure 1D). The secretion of antagonistic factors (both proteases and their inhibitors) by macrophages may relate to the paradoxical effect of removing macrophages on fibrosis: depletion of macrophages can either abrogate fibrosis or enhance it depending on context (Duffield et al., 2005).

Here, we present a cell-circuit framework for tissue repair and fibrosis. This framework clarifies how the interactions between the relevant cell types provide multistability: the dynamics can flow to the different physiological states of fibrosis or healing, depending on the severity and duration of the immune response. This approach builds on previous theoretical work regarding fibroblast activation (Hao et al., 2014) and adds to it the crucial component of the fibroblast interaction with macrophages. The circuit predicts the existence of three steady-states: a state of healing associated with modest ECM production and two fibrosis states associated with high cellularity and excessive ECM production, consistent with histopathological observations. In one of the fibrotic states, which we term “hot fibrosis,” myofibroblasts and macrophages are present at high levels. In the other state, which we term “cold fibrosis,” only myofibroblasts are present. The reciprocal macrophage-myofibroblast interaction is a key component in understanding observations such as the fibrotic time-window, the long timescale of scar maturation, and the paradoxical effect of macrophage depletion. Furthermore, the model suggests several targets for therapeutic reduction of fibrosis, including the PDGF autocrine loop.

## Results

### Macrophages and Myofibroblasts Interact to Form a Multi-Stable Circuit

We developed a circuit framework for the cell-cell interactions in wound healing (Figure 1A). Wound healing involves at least three cell types. The first cell type is the tissue parenchymal cells, such as epithelial cells, that are damaged, and may eventually regenerate. These cells provide signals that recruit and activate the two other cell types: macrophages derived from circulating monocytes and myofibroblasts derived from fibroblasts.

The presence of three cell types requires a three-cell circuit. We find that a considerable simplification occurs if we focus on only two of the cell types, macrophages and myofibroblasts, and consider the tissue parenchymal cells as a source of inflammatory signal that lasts as long as damaged cells are present. We thus consider the damage signal as an input that we can vary to explore transient versus prolonged injury. The resulting two-cell circuit approach allows a clear understanding of the dynamics. In the Supplemental Information we analyze the full three-cell circuit framework and find that it leads to the same essential conclusions (Figure S1).

The two cell types, myofibroblasts and activated wound macrophages, communicate by secreting and sensing growth factors that are essential for their survival and promote proliferation. Myofibroblasts secrete CSF for the macrophages. Macrophages secrete PDGF for the myofibroblasts, as do the myofibroblasts themselves in an autocrine loop (Bonner, 2004). These growth factors are primarily removed via endocytosis by their target cells (Zhou et al., 2018). Here, we focus on monocyte-derived macrophages and include their different states (such as M1 and M2 states) into a single variable.

We assume that myofibroblasts are close to their *carrying capacity*—the maximum population size that can be supported in the tissue. In contrast, macrophages are far from their carrying capacity, as evidenced by the sharp rise in immune cell numbers during inflammation (Figure 1C). A similar situation was found in a study of tissue homeostasis, where fibroblast carrying capacity was measured *in vitro* (Zhou et al., 2018).

We use this circuit to mathematically model the dynamics at the site of an injury. The variables are the numbers of myofibroblasts and macrophages at the injury site. The model takes into account secretion and consumption of growth factors as well as proliferation and removal of cells (Transparent Methods Equations 1–4). For simplicity, we use a single growth factor variable per cell type, which represents an aggregate of multiple ligands that can bind a single receptor as in the case of IL-34 and CSF-1 for CSF1R (Preisser et al., 2014).

We used biologically plausible rate constants for secretion, endocytosis, proliferation, and apoptosis based on experimental measurements (Table 1). We consider these parameter values as a reference set of parameters, and note that different tissues can show different parameter sets.

**Table 1:** Model Parameter Values

Parameter	Biological Meaning	Value	Ref.
*λ*_1_	Maximal proliferation rate of myofibroblasts	0.9 *day*^−1^	(Zhou et al., 2018)
*λ*_2_	Maximal proliferation rate of macrophages	0.8 *day*^−1^	(Zhou et al., 2018)
*μ*_1_, *μ*_2_	Removal rate of the cells	0.3 *day*^−1^	(Zhou et al., 2018)
*K*	Myofibroblast-carrying capacity	10^6^*cellsper ml*$\left(∼10^{-6}\frac{cell}{\mu m^{3}}\right)$	(Zhou et al., 2018)
*k*_1_, *k*_2_	Binding affinity (*K*_*d*_) of growth factor *C*_*ij*_	10^9^*moleculesper ml*	(Hayashida et al., 1990, Nakoinz et al., 1990)^a^
*β*_1_	Maximal secretion rate of CSF1 by myofibroblasts	$470\;\frac{molecules}{cell\;min}$	BNID 112718
*β*_2_	Maximal secretion rate of PDGF by macrophages	$70\;\frac{molecules}{cell\;min}$	BNID 112718
*β*_3_	Maximal secretion rate of PDGF by myofibroblasts	$240\;\frac{molecules}{cell\;min}$	BNID 112718
*α*_1_	Maximal endocytosis rate of CSF1 by macrophages	$940\;\frac{molecules}{cell\;min}$	BNID 112725
*α*_2_	Maximal endocytosis rate of PDGF by myofibroblasts	$510\;\frac{molecules}{cell\;min}$	BNID 112725
*γ*	Degradation rate of growth factors	2 *day*^−1^	(Zhou et al., 2018)

BNID, BioNumbers ID number.

a These parameters were adjusted to consider spatial effects.

**Figure 2:**
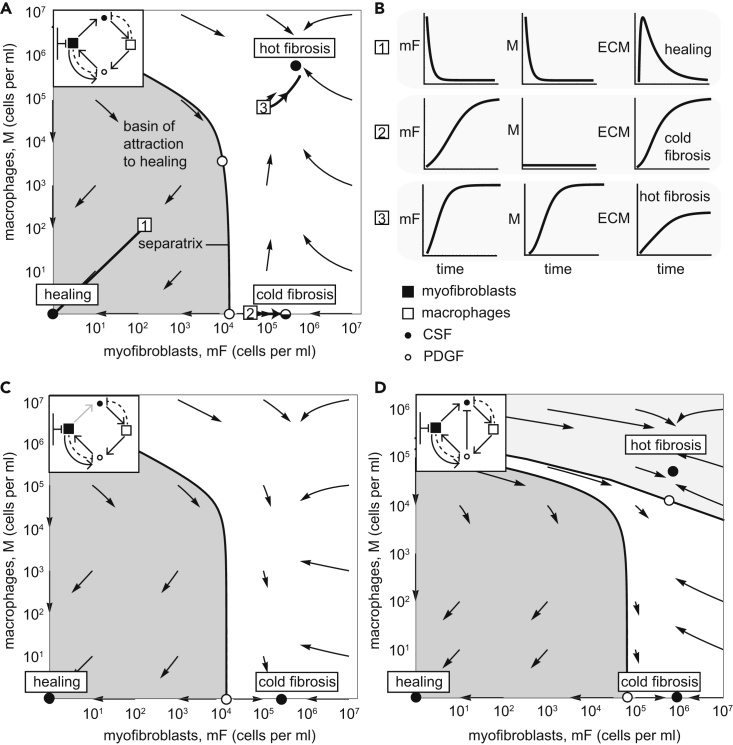
The Myofibroblast-Macrophage Circuit Shows Multi-stability between a Healing State and Two Fibrosis States (A) Phase portrait of the circuit with reference parameters, in which arrows show the flow rate of change of cell numbers. Stable fixed points (black dots) and unstable fixed points (white dots) are shown (the semi-stable cold fibrosis state as split black and white), as is the separatrix (black line) that marks the boundary between the basin of attraction of the healing (gray region) and fibrosis states (the parameter values that are used are listed in Table 1). (B) Temporal trajectories of cells and ECM starting from the initial points 1–3 (white squares in panel B). (C) Phase portrait of a circuit with a 100-fold lower CSF secretion rate than in panel A stabilizes the cold-fibrosis state. There is no hot fibrosis state. (D) Myofibroblast-macrophage phase portrait in a circuit in which PDGF downregulates CSF expression in myofibroblast shows all three stable states. Note there are two separatrix curves, dividing the phase portrait into three basins of attraction. The middle basin (white region) flows to the cold-fibrosis state. Here we used the parameter values: $\alpha_{1}=0.2\frac{molecules}{cell\;min}$, $\alpha_{2}=30\frac{molecules}{cell\;min}$, $\beta_{3}=8\frac{molecules}{cell\;min}$, $\beta_{1}=2\frac{molecules}{cell\;min}$, $\lambda_{1}=\lambda_{2}=2\frac{1}{day}$. We used the values listed in Table 1 for the remaining model parameters. See also Figure S2.

The resulting dynamics can be displayed in a *phase portrait*, in which the axes are the concentrations of the two cell types (cells per ml of tissue volume). The arrows show the direction of change of the cell concentrations (Figure 2A).

The model shows three types of dynamic processes. At low initial cell (myofibroblast + macrophage) concentrations, the dynamics flows toward a stable state with zero cells (Figure 2A). This state can be considered as the healing state, because the scarring process is resolved when monocyte-derived macrophages and myofibroblasts are removed from the tissue, allowing the tissue-resident cell types to proliferate and restore tissue homeostasis.

However, if the initial cell numbers are high enough, above a threshold denoted by the *separatrix* line in Figure 2A, the dynamics flows to a steady state with high levels of both cell types. This steady-state is maintained with constant turnover of the cells. Myofibroblasts and macrophages keep each other at a high concentration due to the mutual secretion of growth factors. We consider this stable state as a “hot fibrosis” state because both myofibroblasts and macrophages linger within the tissue and there is constant production of ECM. The term “hot” indicates the high abundance of immune cells (macrophages).

The model also has a third fixed point. In this fixed point, there are zero macrophages and a high level of myofibroblasts. This state leads to ECM production because of the high level of myofibroblasts and may be considered as another state of fibrosis. We term this “cold fibrosis” due to the lack of macrophages (Figure 2A).

The hot fibrosis and the healing fixed points are stable for a wide range of model parameters: the reference parameter values can vary by 10- to 100-fold without affecting the stability of the healing and hot fibrosis states (Figure S2). The cold fibrosis state is robustly stable to changes in myofibroblast numbers but unstable regarding changes in the abundance of macrophages. A small influx of macrophages causes propagation to the hot fibrosis state (Figure 2A).

The cold fibrosis state can, however, be made fully stable by using different values for the model parameters. For example, a 100-fold lower CSF secretion rate causes the hot fibrosis state to lose its stability, leaving only the healing and cold fibrosis states as stable solutions. In this case, perturbing cold fibrosis by adding macrophages leads to a return to cold fibrosis and a removal of the macrophages (Figure 2C).

Another scenario that stabilizes the cold fibrosis state is considering a slightly modified model in which CSF production in myofibroblasts is downregulated by PDGF (Transparent Methods Equation 5). In this case, perturbing cold fibrosis by adding a small amount of macrophages leads to a return to cold fibrosis and a loss of the macrophages; addition of a large amount of macrophages causes flow to the hot fibrosis state (Figure 2D). The phase portrait shows three basins of attraction: to healing, cold, and hot fibrosis.

These scenarios with different choices of model parameters or additional interactions may exemplify the circuit in different tissue contexts. Indeed, different organs can have different patterns of fibrosis. Carrying capacity of either cell type may be different in different tissue types, limited by anatomy (space), physiology (e.g. oxygen supply), or characteristics of the surrounding cell populations.

To chart the amount of ECM during these dynamics, we computed the accumulation of ECM produced by myofibroblasts (Figure 1D). We considered ECM degradation by proteases including MMPs, and the inhibition of proteases, by factors including TIMPs (see Transparent Methods Equations 6–12 and parameter values in Table 2). The healing state has a small amount of ECM accumulation followed by degradation, whereas the hot fibrosis and the cold fibrosis states have high persistent level of ECM (Figure 2B).

**Table 2:** Dimensionless Parameter Values for ECM Dynamics

Parameter	Definition	Value
*a*	$a\frac{\gamma k_{21}}{\beta_{21}k_{E}\alpha_{A}}$	1
*b*	$b\frac{K}{k_{E}\alpha_{A}}$	1
*c*	$c\frac{\gamma k_{21}}{\beta_{21}k_{E}\alpha_{P}}$	1
*P*_0_	$\frac{P_{0}}{k_{E}\alpha_{P}}$	0.1
*α*_*E*_	$\frac{\alpha_{E}}{\mu_{1}}$	1

Although we model ECM concentration, the same variable may be interpreted to include also matrix quality such as stiffness (Klingberg et al., 2013). Often, excess matrix is stiffer, abnormal, and partially degraded. Such stiffness may accelerate myofibroblast differentiation and accumulation, as well as enhance the transition to fibrosis (Avery et al., 2018).

### Bistability of the Circuit Explains How Persistent Injury Triggers Fibrosis

To study the effect of the duration of injury on fibrosis, we considered the response of the circuit to several scenarios: injury stimuli consisting of a single transient pulse, repetitive injury pulses, or a single prolonged pulse (we use the reference parameter set; we examine the response of the circuits with a stable cold fibrosis state in the Supplemental Information (Figure S3)). We modeled these injury stimuli by inflammatory signals that recruit monocyte-derived macrophages and thus serve as a source term in the macrophage equation (Transparent Methods Equations 13 and 14).

We numerically solved the dynamical trajectory of the cells in response to these injuries starting from an initial condition of a small number of macrophages and myofibroblasts (1 cell/mL). The results do not depend sensitively on this initial condition. We find that the outcome of the cell-circuit response depends on the severity, duration, and recurrence of the injury.

**Figure 3:**
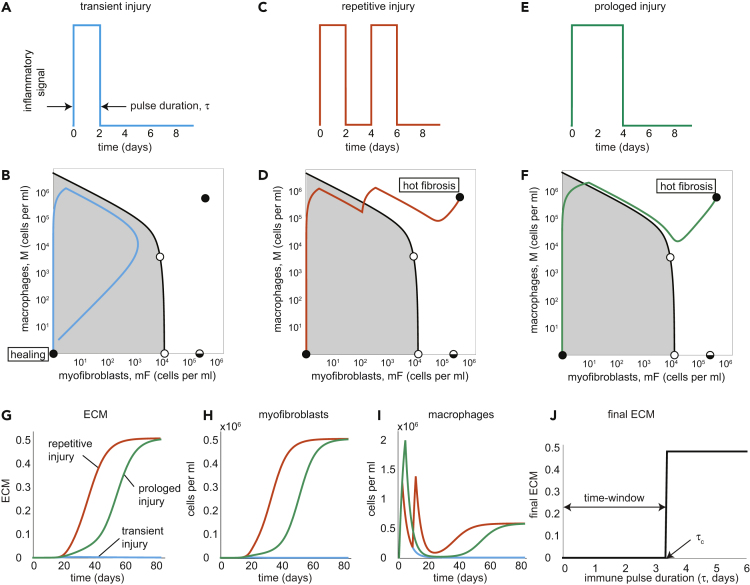
The mF-M Circuit Shows Healing Versus Fibrosis Depending on Duration and Recurrence of Inflammation (A–I) (A) For a brief pulse of inflammation (2 days), the rise of mF and M is transient, leading to a healing trajectory in phase space that returns to the healing state (in light blue) (B). In contrast, two successive two-day long inflammatory pulses (C) or a prolonged pulse (4 days) (E) lead to a trajectory to the hot fibrosis state with persistent mF and M populations (in orange and green, respectively) (D and F). Note that the separatrix applies to the equations without the external inflammation input, and so the separatrix can be crossed during the input pulse(s). The dynamics of ECM (G), mFs (H), and Ms (I) are plotted in response to transient (light blue lines), repetitive (orange lines), and prolonged (green lines) injuries. (J) Final ECM as a function of inflammation pulse duration shows a critical time-window of about three days to stop inflammation. See also Figures S1, S3, and S4.

A transient injury stimulates a brief inflammatory pulse (Figure 3A) that recruits a strong, brief influx of macrophages. Then, myofibroblast numbers rise. When the pulse ends, the macrophages die and no longer support myofibroblast proliferation. Both cell types decline to zero and reach the healing stable state (Figure 3B). The overall accumulation of ECM is very small (Figure 3G).

In contrast, a repetitive (Figure 3C) or prolonged injury (Figure 3E) leads to a different result. The cells divide and grow, mutually supporting each other via growth factors, until they cross the separatrix between the healing and fibrosis states. The cells converge to the hot fibrosis state (Figures 3D and 3F). ECM accumulation reaches a high steady-state, which represents scar formation in fibrosis (Figure 3G).

Brief repetitive injuries have some dynamical differences from a single prolonged injury. When the prolonged injury elicits a response that is close to the separatrix, the scar shows a slower accumulation than repetitive injury (Figure 3G, green line compared to orange line) due to the temporary loss of macrophages (Figure 3I). Very prolonged injury becomes more similar to repetitive injury (Figure S4).

To illustrate the existence of a critical time-window, we plot the final ECM level as a function of the duration of a transient immune pulse (duration of inflammation). The final ECM level is very small up to a critical inflammation duration of *τ*_*c*_∼3 days. For longer inflammation, ECM jumps to a high final level, representing fibrosis (Figure 3J). The critical time-window is due to the crossing of the separatrix. Thus, the model predicts that inflammation must be stopped early in order to avoid fibrosis.

### The Timescale of Months for Scar Maturation Is due to the Slow Crossing of a Dynamical Barrier

**Figure 4:**
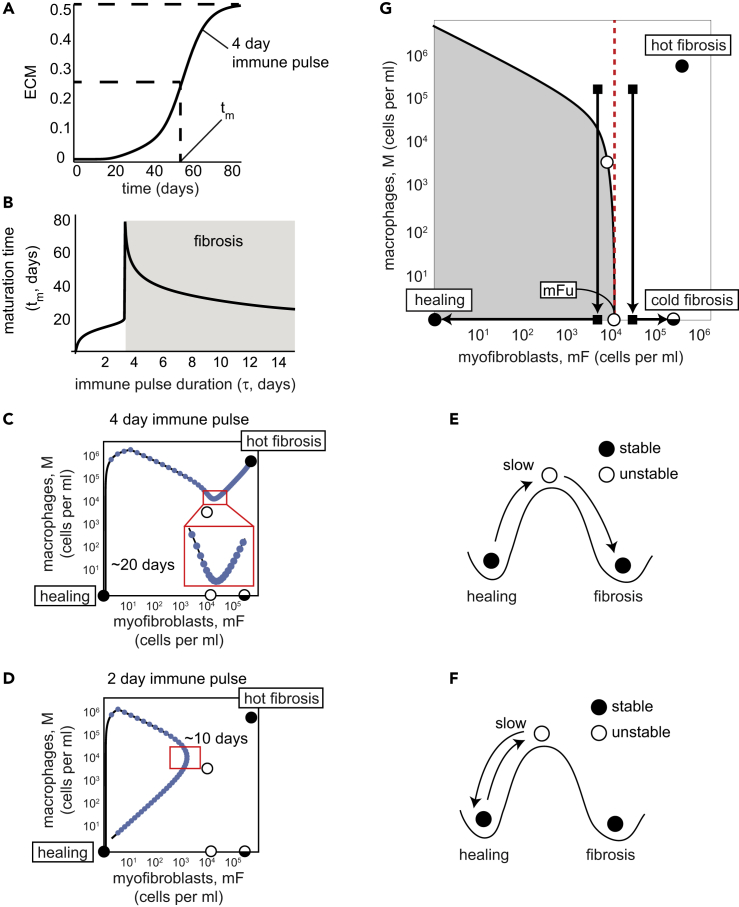
The mF-M Circuit Explains the Timescale of Months for Scar Maturation and the Paradoxical Effect of Macrophage Depletion on Fibrosis (A) ECM accumulation in response to a four-day immune pulse. Maturation time, *t*_*m*_, is defined as the time to reach half maximal ECM accumulation. (B–F) (B) ECM maturation time is on the order of months for fibrosis and weeks for healing. The slow timescale of weeks to months is due to a dynamical barrier due to an unstable fixed point (upper white circle) (C and D), akin to a ball slowing down at the top of a hill (E and F). In (C and D) blue dots indicate trajectory values at intervals of one day, so that slow dynamics correspond to dense blue dots. (G) Depleting macrophages when myofibroblasts are below or above the unstable point (lower white circle, labeled mFu) leads to healing or fibrosis, respectively.

We also analyzed the time to reach full ECM levels, which corresponds to the scar maturation time. We define the maturation time (*t*_*m*_) as the time to reach half of the final ECM level (Figure 4A). The maturation time is on the order of a month and reaches 80 days for immune pulses close to the critical pulse duration (Figure 4B, see Transparent Methods). This month-timescale is surprising, because it is much longer than the turnover time of the growth factors (hours) or the cells (days).

To understand the slow maturation time, we zoomed in on the dynamics for an immune pulse of four days (Figure 4C). During the pulse, there is a sharp rise in macrophages due to influx from monocytes. Because the pulse is brief, there are only a few myofibroblasts when the pulse ends, and they cannot strongly support the macrophages by CSF secretion. Hence the macrophage population drops after the pulse is over but does not vanish.

The dynamics are then close to an unstable fixed point (upper white circle in Figure 4C). Exactly at the fixed point the rates of change are, by definition, zero. Therefore, close to the fixed point the rates of change are small, and the system slowly moves away from the fixed point by means of cell growth. Intuitively, the tissue crosses a dynamical threshold from the healing state to the hot fibrosis state, akin to a ball that slows down when it reaches the peak of a hill (an unstable fixed point), from which it descends to a new fixed point (Figure 4E). Indeed, the dynamics creep up slowly toward the hot fibrosis state, but it takes about 20 days to appreciably move toward this mature final state (Figure 4C).

Interestingly, this slow timescale is also seen when healing occurs in the model. For an immune pulse of two days (Figure 4D), which is shorter than the critical window, the dynamics also come close to the unstable fixed point, but in this case the trajectory drops back to the “healing” side of the hill (Figure 4F). Indeed, experiments show that non-fibrotic resolution of transient injury can take weeks (Seifert et al., 2012).

### Depletion of Macrophages at Different Myofibroblast Numbers Show Opposing Effects

We next analyze the effect of macrophage depletion, which has been reported to be either pro- or anti-fibrotic in different experiments. Once macrophages are depleted, myofibroblast survival depends on their autocrine loop. This autocrine loop provides bistability in which myofibroblasts either converge to the cold fibrosis state and survive or die and cause the tissue to flow to the healing state. The threshold for myofibroblasts, above which they can sustain their proliferation without macrophages, is the unstable fixed point mFu (lower white circle in Figure 4G).

This unstable fixed point can explain the paradoxical effects of removing macrophages on fibrosis. If macrophages are removed early enough, before myofibroblasts cross mFu, the myofibroblasts cannot support themselves, and the system flows to the healing state. In contrast, removing macrophages when myofibroblasts exceed mFu leads to a rapid flow to the cold fibrosis state characterized by high ECM (Figure 4G). This bistability may explain why removal of macrophages enhanced healing in some studies, whereas in other studies, removal led to accelerated fibrosis (Duffield et al., 2005), even though removal started at similar levels of macrophages.

### Weakening the PDGF Autocrine Loop or Slowing Myofibroblast Proliferation May Prevent or Reverse Fibrotic Response

**Figure 5:**
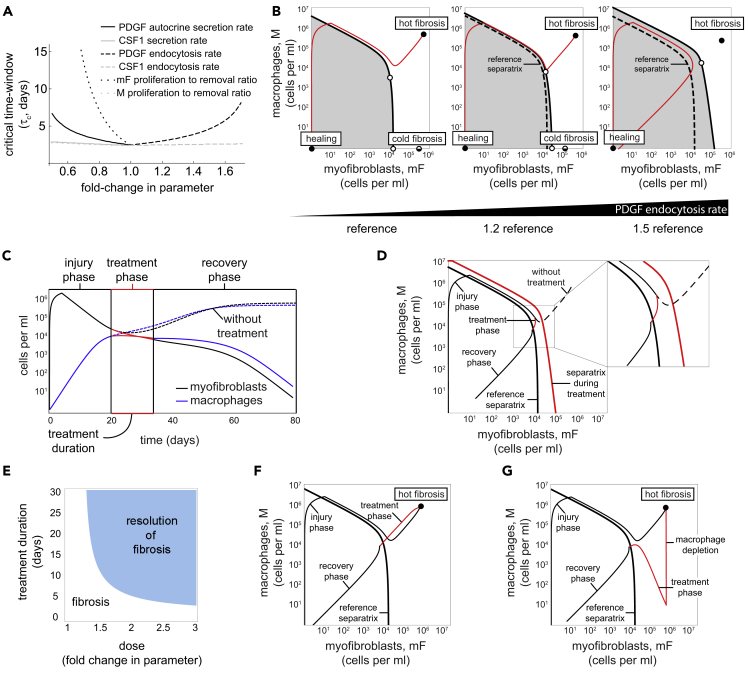
Fibrosis Can Be Prevented or Reversed by Changing Several Circuit Parameters (A) Critical time window for inflammation that results in healing as a function of fold-change in circuit parameters. The window can be lengthened by decreasing autocrine secretion, increasing PDGF endocytosis, or decreasing the ratio of mF proliferation to removal. (B) Increasing PDGF endocytosis rate by 150% eliminates the cold fibrosis state, enlarging the basin of attraction to the healing state (gray region) and allowing a four-day immune pulse to resolve in healing. (C) Dynamics of myofibroblasts (black) and macrophages (blue) following a four-day immune pulse that lead to fibrosis without treatment (dashed curves). A 14-day treatment in which the PDGF endocytosis rate is increased by 150% that is given 20 days after the injury leads to healing. (D) The same 14-day treatment is shown in a phase portrait of the cells. The timing of the treatment is given when the cells are below the separatrix with the altered parameters (in red), and the duration of the treatment lasts until the cells cross the original separatrix (in black). (E) The effect of changing the PDGF endocytosis rate 20 days following a four-day immune pulse is shown for different values of duration and dose of the treatment. (F) Reversing a mature scar following a four-day immune pulse by increasing PDGF endocytosis rate by 300% for 40 days. (G) Reversing the same mature scar by a 25-day treatment of 300% larger PDGF endocytosis rate following macrophage depletion.

Finally, we asked what interventions might help to prevent fibrosis. Recent efforts to find anti-fibrotic drugs focus on growth factor inhibitors (for example, CSF1- or CSF1R-targeted therapeutic antibodies) or kinase inhibitors such as Nintedanib (Bellamri et al., 2019, Doloff et al., 2017, Hostettler et al., 2014, Meziani et al., 2018). To find conditions in the cell-circuit framework that can reduce the chance of fibrosis, we varied each of the model parameters around the reference parameter values and computed the duration of injury signal that is the tipping point to fibrosis (the duration of the time-window). We find that changing four of the parameters can lengthen the time-window and therefore make fibrosis more difficult to achieve. These parameter changes are a decrease in PDGF autocrine secretion (*β*_3_), an increase in PDGF endocytosis rate (*α*_2_), a decrease in myofibroblast proliferation rate (*λ*_1_), or an increase in their removal rate (*μ*_1_) (Figure 5A, see Transparent Methods).

The effect of these changes is to enlarge the basin of attraction for the healing state. Such enlargement means that more situations end up resolved without fibrosis. We find that a large increase in the basin of attraction occurs in the model when the cold fibrosis fixed-point vanishes. The requirement for removal of the fixed point, and hence a decrease in the likelihood of fibrosis, can be summarized as a single requirement on a dimensionless combination of the four parameters mentioned above: *C* = *β*_3_*λ*_1_/*α*_2_*μ*_1_needs to be smaller than 1, as can be analytically shown (see Transparent Methods Equations 15–19). The cold fibrosis state vanishes also for a larger PDGF degradation rate (*γ*) or a larger binding affinity of PDGF to its cognate receptor (*k*_2_) (Figure S2).

The dynamics when this condition is met (*C* = 0.9 and thus *C*< 1) are shown in Figure 5B. A relatively long immune pulse of four days, which would lead to fibrosis with the reference parameter values, now flows to the healing state with no fibrosis (Figure 5B). The hot fibrosis state can still be reached after a pulse of six days.

The intervention to prevent fibrosis can be short term: once the reference separatrix is crossed and the basin for the healing state is entered, the circuit will flow to the healing state even if one stops the treatment (Figures 5C and 5D). For example, treating an injury that elicits a four-day immune pulse by weakening the PDGF autocrine loop for 14 days starting 20 days after the injury results in removal of the cells and resolution of fibrosis (Figure 5C). The larger the dose of the treatment (larger dose is modeled as a larger change in the model parameter), the shorter the duration of the treatment needed to prevent fibrosis (Figure 5E).

We also asked whether fibrosis can be reversed, in the sense that a mature scar in the hot fibrosis state can be made to flow to the healing state. This can be achieved in two ways. The first option is to use an intervention that achieves the criterion C < 1 on the model parameters such that the hot fibrosis state loses its stability and the only stable state is the healing state (Figure 5F). The second approach is to combine the first approach with macrophage depletion. In this second approach one may reverse a mature scar by first depleting macrophages and then treating with an intervention that eliminates the cold fibrosis state (Figure 5G). In both of the approaches to reverse fibrosis the treatment can be short-term; using macrophage depletion shortens the required treatment duration by almost two-fold. This predicted reversal of fibrosis depends on the fact that fibrosis is a dynamic steady-state with cell turnover.

## Discussion

**Figure 6:**
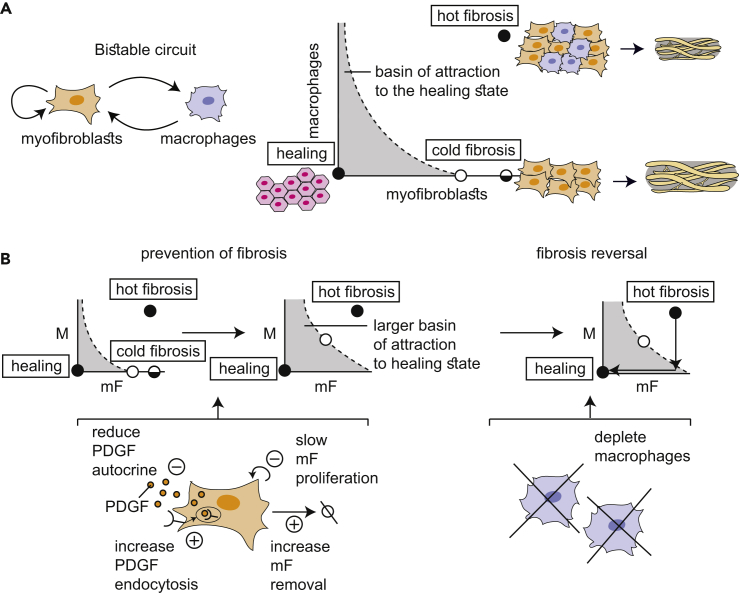
Overview of the Present Circuit Approach to understanding Healing and Fibrosis (A) The mF-M circuit shows a stable healing state and two fibrosis states. Outcome depends on the duration and persistence of inflammation pulses caused by an injury, which can remain in the basin of attraction for healing, or cross the separatrix into the basin of attraction for fibrosis. (B) The present analysis suggests targets for prevention and reversal of fibrosis, by eliminating the cold fibrosis fixed point and enlarging the basin of attraction to the healing state. See also Figure S5.

This study presented a circuit for wound healing and fibrosis based on the interactions between myofibroblasts and inflammatory macrophages. The circuit explains how two different outcomes result from the same system: healing when injury is transient and fibrosis when the injury is prolonged or repetitive. Each of these outcomes has its own basin of attraction in a bistable or multistable phase diagram (Figure 6A). We use the cell-circuit framework to explain several physiological and pathological phenomena in tissue repair and fibrosis. First, we show that the cell-circuit response depends on the duration of the inflammatory signal, which explains the origin of a brief critical time-window in which removing inflammation abrogates fibrosis. Second, we show that the months-long timescale for scar maturation, which is surprising given the much shorter timescales of cell signaling and proliferation (hours to days), originates from a slowdown near an unstable fixed point. Thirdly, we explain the paradoxical effect of macrophage depletion: healing or fibrosis occurs when myofibroblasts are below or above a critical threshold, respectively, where they can support their own proliferation at the time of macrophage depletion. Finally, we suggest potential targets for preventing and reversing fibrosis including the PDGF autocrine loop.

The present circuit framework indicates that constant cell turnover is needed to maintain fibrosis states. It explains why several types of fibrosis are sometimes seen in different clinical settings (Soderblom et al., 2013). The circuit shows two types of fibrosis, hot fibrosis with both macrophages and myofibroblasts and cold fibrosis with only the latter (Figure 6A). The cold fibrosis fixed point explains why the depletion of macrophages can sometimes result in accelerated fibrosis (Duffield et al., 2005).

Parallels can be drawn to several pathologies. Hot and cold fibrosis may correspond to the tissue composition of pathological scars in dermatology. The two main types of pathological scars in the skin are hypertrophic scars and keloid (Abergel et al., 1985, Cohen et al., 1972, Craig et al., 1975, Ogawa, 2017, Trace et al., 2016). Keloids are characterized by high densities of macrophages, as in hot fibrosis, and inflammation persists over years (Santucci et al., 2001). In contrast, the transition from early to late hypertrophic scars is accompanied by a progressive decrease of immune cell infiltrates, as in cold fibrosis (Santucci et al., 2001).

Keloids (hot fibrosis) can be treated by anti-proliferative therapies such as local injection of cortisol, cryotherapy, radiotherapy, or topical application of cytostatic drugs (Arno et al., 2014). An exclusive surgical treatment of keloids results in regrowth (Love and Kundu, 2013), in contrast to hypertrophic scars. Based on the model, moving from hot fibrosis to cold fibrosis by a cytostatic treatment that affects activated macrophages brings the system to a state closer to the cold fibrosis of hypertrophic scars; this can be followed by surgery. This conclusion is coherent with the observation that combining keloid surgery with anti-proliferative treatment decreases recurrence (Arno et al., 2014).

Hot fibrosis may also characterize fibrotic diseases caused by persistent damaging agents, such as pneumoconiosis or liver fibrosis. These fibrotic lesions typically show infiltrates of myofibroblasts as well as macrophages (Castranova and Vallyathan, 2000, Lech and Anders, 2013, Pellicoro et al., 2014, Tesch, 2010), which resembles hot fibrosis. Analysis of the basins of attraction of a model with stable cold fibrosis suggests that very prolonged injury, such as damage signals generated by damaging agents that cannot be removed, leads to hot fibrosis (Figure S3B). Injury signals of intermediate duration would tend to lead to cold fibrosis (Figure S3B).

The present minimal circuit approach suggests that bistability is a core mechanism of wound healing and fibrosis. Minimal models allow us to reveal design principles that are at the core of biological phenomena as was demonstrated in other biological contexts (Alon, 2019). The analysis and reference parameters are based on *in vitro* experiments with mouse cells (Zhou et al., 2018). Thus, it is likely that there will be mismatches between the model and measurements in human tissue contexts. An understanding of multiple tissue processes in response to injury including spatial effects and chemokine gradients, surrounding cell populations, additional parenchymal cell damage, and upstream signaling pathways that converge into fibrosis such as TGF-β (Leask and Abraham, 2004, Lodyga et al., 2019) is required to scale this approach to the complexity found in human disease. Each cell-type has multiple subtypes (such as M1 and M2 macrophages) and sometimes even a continuum of states (Adler et al., 2019, Korem et al., 2015). This diversity is being revealed with single-cell analysis of fibrosis (Dobie and Henderson, 2019). Fibrosis itself can cause additional parenchymal cell damage, for example by broadening the distance between blood vessels and parenchymal cells leading to hypoxic conditions, or by causing stiffness in organs that need flexible and motile elements for normal function. Such processes will require extensions of the present model. They will impact the circuit parameters such as carrying capacities, cell growth and removal rates, and growth-factor production rates. Analyzing the circuit behavior with different parameters and additional interactions might expand the applicability of the present minimal circuit model. We hypothesize that in many of these extensions, bistability or multistability will remain at the heart of the transition between healing and fibrosis.

It would be interesting to explore how the myofibroblast-macrophage circuit might interface with cancer, to model the fibrotic-like microenvironment that promotes cancer incidence and cancer growth (Dvorak, 1986, Kalluri, 2016, Marsh et al., 2013). The model can also be used to explore the role of senescent cells in healing and fibrosis, where at young ages senescent cells enhance healing, whereas they seem to increase the chances for fibrosis at old ages (Figure S5).

The present circuit suggests possible therapeutic targets for fibrosis, such as weakening the PDGF autocrine loop for myofibroblasts. Conversely, enhancing PDGF autocrine secretion rate is predicted to worsen fibrosis. Indeed, overexpressing PDGFα in mouse myofibroblasts increased the probability of fibrosis (Olson and Soriano, 2009). In the present model, fibrosis can be prevented by any of the following or their combination: reduction of PDGF autocrine secretion rate, reduction of myofibroblast proliferation rate, increase in PDGF endocytosis or degradation rate, increase in PDGF binding affinity, or increase in myofibroblast removal rate. Fibrosis can also be reversed according to the present picture. To reverse fibrosis requires altering any of the above parameters, in order to abrogate the fibrosis fixed points, together with depletion of macrophages from the mature scar, to cause the system to flow to the healing state (Figures 5F and 5G).

### Limitations of the Study

This study presents a model of a minimal circuit for tissue repair and fibrosis based on growth-factor interactions. The model excludes several tissue processes that may be important to consider in order to expand the scope of the model's agreement with the physiological behavior of fibrotic tissues. These processes include stochastic effects, spatial effects and chemokine gradients, surrounding cell populations (e.g. tissue resident fibroblasts, macrophages and parenchymal cells), and interactions between the ECM and the cells.

## Methods

All methods can be found in the accompanying Transparent Methods supplemental file.

## Data and Code Availability

The code that generates all the figures in this study is available in GitHub, at https://github.com/AlonLabWIS/fibrosis-cell-circuit.

## Supplemental Information

Document S1. Transparent Methods and Figures S1–S5

## Footnotes

Supplemental Information can be found online at https://doi.org/10.1016/j.isci.2020.100841.
