# Supplementary material for: Principles of Cell Circuits for Tissue Repair and Fibrosis
Source: iScience. 2020 Jan 16;23(2):100841. doi: 10.1016/j.isci.2020.100841 (PMC7005469; doi:10.1016/j.isci.2020.100841)
Supplement: Document S1. Transparent Methods and Figures S1–S5 [file mmc1.pdf]

iScience, Volume *23*

Supplemental Information

Principles of Cell Circuits

for Tissue Repair and Fibrosis

Miri Adler, Avi Mayo, Xu Zhou, Ruth A. Franklin, Matthew L. Meizlish, Ruslan Medzhitov, Stefan M. Kallenberger, and Uri Alon

Supplementary Information

Myofibroblasts, damaged epithelial cells, and inflammatory macrophages interact to form a multi-stable circuit

We model the interactions between damaged epithelial cells (D), myofibroblasts (mF), and macrophages (M) using the same equations for myofibroblasts and for the growth factors as in the Transparent Methods section (Eqs. 1, 3-4) and the following equations for macrophages and the damaged epithelial cells:

(S1) $\dot{M} = D + M\left(\lambda_2 \frac{CSF}{k_2+CSF} - \mu_2\right)$

(S2) $\dot{D} = d(t)(N - D) - \alpha\, D$

where $d(t) = d_0\big(\theta(t) - \theta(t-\tau)\big)$ is the damage stimulus, N is the total concentration of epithelial cells in the tissue including normal and damaged cells, and α is the removal rate of the damaged epithelial cells. In Eq. S1, the term D represents the factors secreted by the damaged cells that recruit monocytes/macrophages.

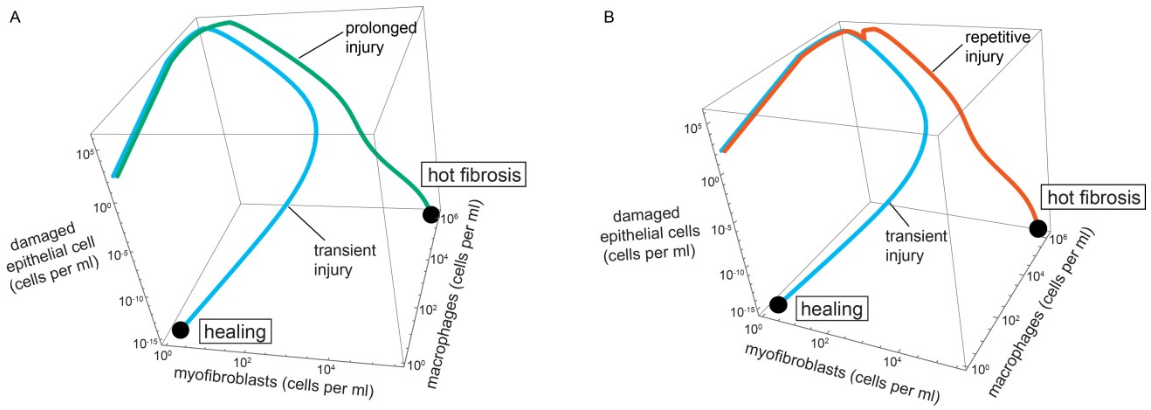

Figure S1: A three-cell-type circuit of communicating myofibroblasts, damaged epithelial cells, and inflammatory macrophages shows healing versus fibrosis depending on duration and recurrence of injury, related to Figure 3. (A) A transient injury that causes damage to epithelial cells leads to accumulation of myofibroblasts and macrophages followed by their removal and removal of damaged epithelial cells (light blue trajectory). In contrast, prolonged injury (green trajectory, A) or repetitive injury (orange trajectory, B) causes the circuit dynamics to flow towards the hot fibrosis state with high levels of myofibroblasts and macrophages, producing large amounts of ECM. We used the parameter values: $N = 10^6\ cells$, $\alpha = 1\frac{1}{day}$, $d_0 = 100\frac{cells}{day}$.

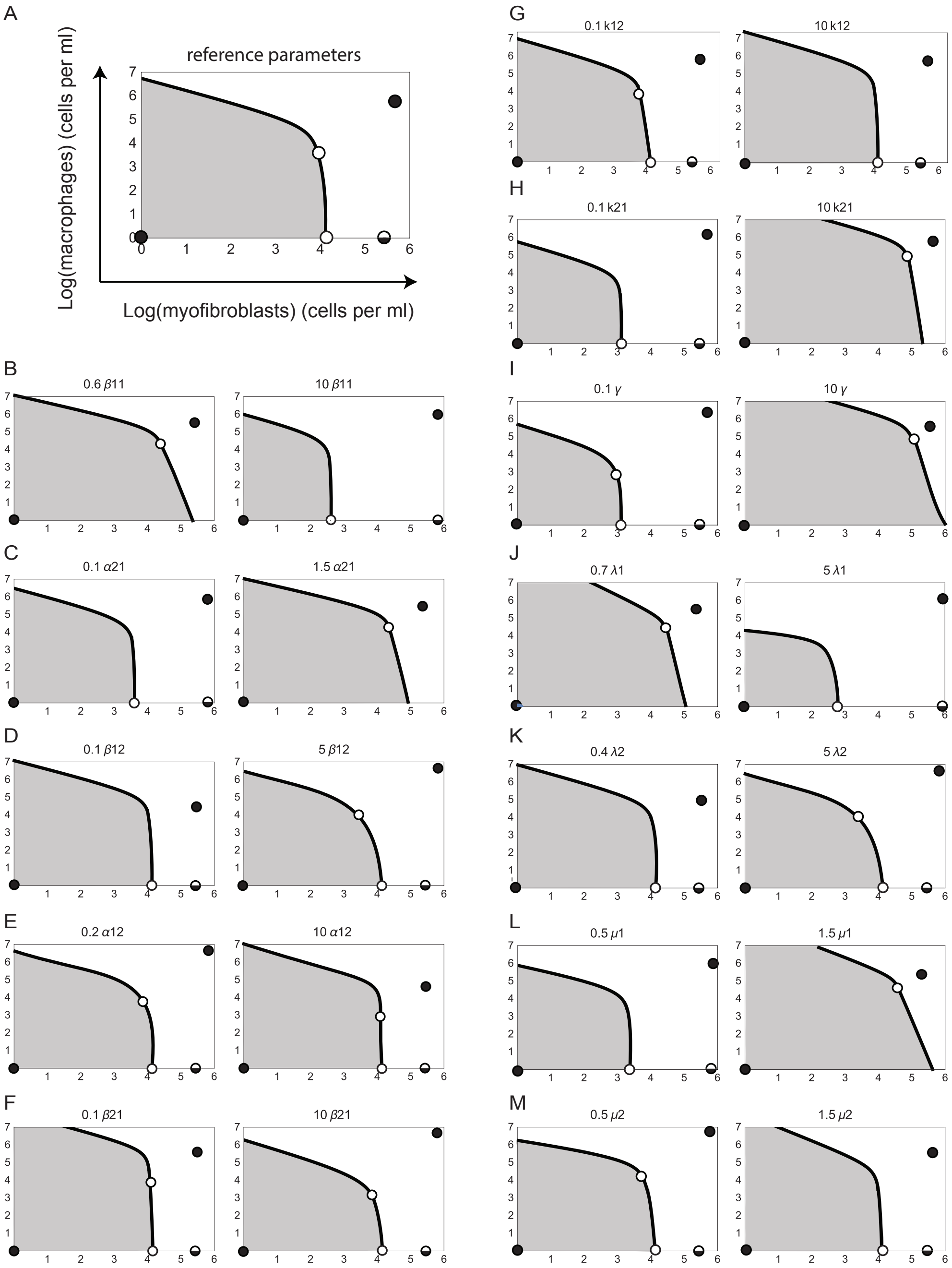

Figure S2: The healing and hot-fibrosis states are stable for a wide range of model parameters, related to Figure 2. (A) Phase portrait with the reference parameter values from Table 1. Stable fixed points (black dots) and unstable fixed points (white dots) are shown (the semi-stable cold fibrosis state is split black and white), as is the separatrix (black line) that marks the boundary between the basin of attraction of the healing state (gray region) and hot-fibrosis state. (B-M) Phase portrait with one of the model parameters smaller (left panels) or larger (right panels) than the reference model parameter value in Table 1. When parameters are shifted by values other than 0.1-fold or 10-fold, the shift indicates the boundary of the stable parameter range.

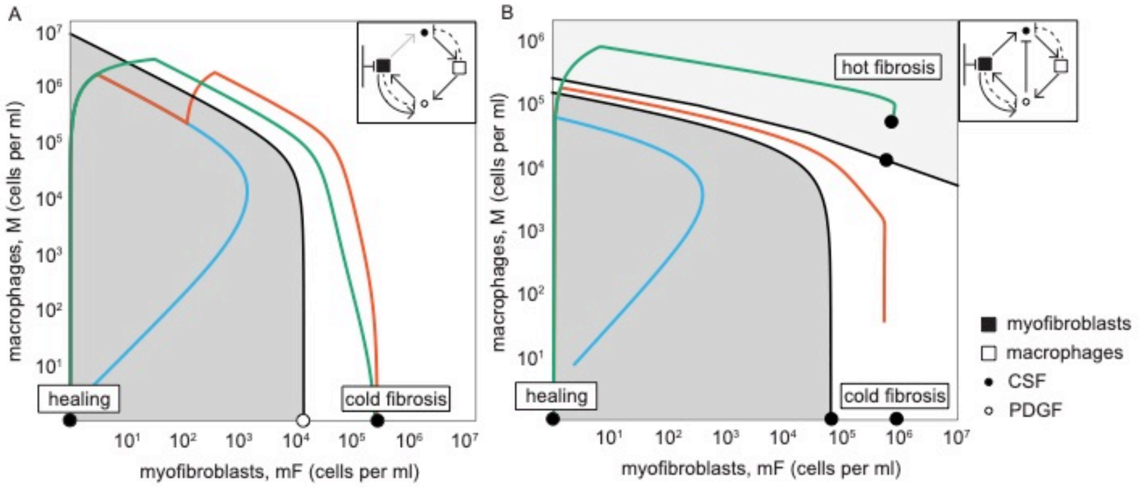

Figure S3: The circuits with a stable cold fibrosis state show healing versus fibrosis depending on duration and recurrence of inflammation, related to Figure 3. (A) Phase portrait of the mF-M circuit with a 100-fold lower CSF secretion rate than the reference parameter value, providing stable healing and cold-fibrosis states. A transient injury leads to the healing state (light blue line), a repetitive (orange line) or a prolonged injury (green line) leads to the cold fibrosis state. (B) Phase portrait of a mF-M circuit in which PDGF downregulates CSF expression in myofibroblast shows all three stable states. A transient injury leads to the healing state (light blue line), an injury with intermediate duration leads to the cold-fibrosis state (orange line), and a prolonged injury leads to the hot-fibrosis state (green line).

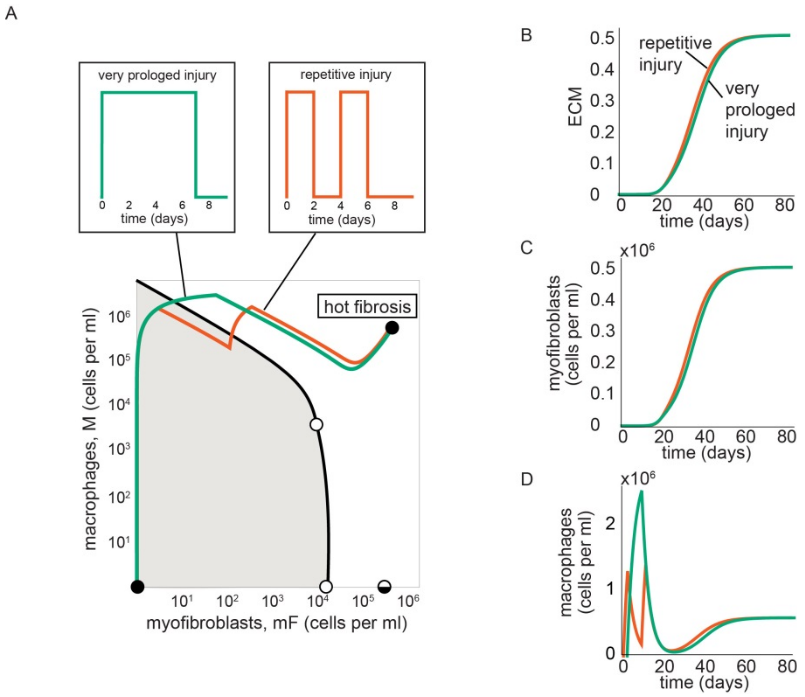

Figure S4: Repetitive injury results in a similar response as a very prolonged injury, related to Figure 3. (A) A 7-day inflammatory pulse and two successive 2-day pulses lead to a trajectory to the hot fibrosis state with persistent mF and M populations (in green and orange, respectively). The dynamics of ECM (B), myofibroblasts (C), and macrophages (D) following these injuries are similar especially at late times. Reference parameters are used.

The model suggests different roles for senescent cells in young and old organisms

We can use the present approach to explore the impact of cellular senescence on tissue repair and fibrosis. Senescent cells (SnCs) are known to be pro-healing at young ages; for example, removing senescent cells impairs repair in the skin and liver (Jun and Lau, 2010; Krizhanovsky et al., 2008). On the other hand, senescent cells are pro-fibrosis at old ages and in age-related diseases. For example, removing whole-body SnCs helps reduce fibrosis in IPF models and in systemic sclerosis (Hecker et al., 2014; Muñoz-Espín and Serrano, 2014; Piera-Velazquez and Jimenez, 2015). The present model can offer a framework to understand this age-dependent role.

SnCs are cells that stop dividing and secrete factors known as senescent-associated secretion profiles (SASP) that includes inflammatory signals, ECM degradation factors and factors that inhibit proliferation of nearby cells (Campisi, 2005; van Deursen, 2014; Rodier and Campisi, 2011). The number of SnCs increases dramatically at old age in many tissues. At all ages, senescent fibroblasts are generated at injury sites during the process of normal healing.

One may propose the following picture: at young ages, systemic SnC levels are low. SnCs are generated locally after injury, including senescent myofibroblasts. They enhance healing because they i) slow down myofibroblast proliferation by a bystander effect through SASP, ii) increase myofibroblast loss due to myofibroblast senescence and iii) degrade ECM. All of these factors enlarge the basin of attraction of the healing state (Fig S5A).

In contrast, at old ages, SnCs are abundant in many tissues even without injury (Campisi, 2005;

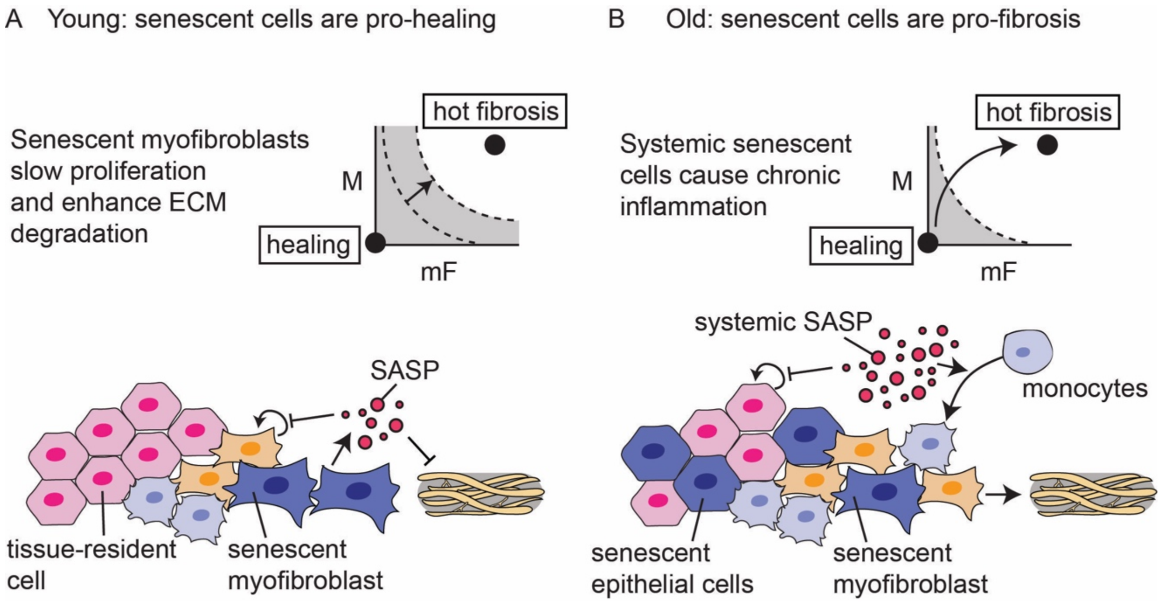

Figure S5: Paradoxical effect of senescent cells on fibrosis, related to Figure 6. (A) Role of SnCs is pro-healing in young individuals in which SnCs are mainly locally generated myofibroblasts at the injury site that enlarge the basin of attraction to the healing state. (B) At old ages, systemic SnCs cause chronic inflammatory signals which can cause the tissue to cross the separatrix.

Muñoz-Espín and Serrano, 2014), including senescent epithelial cells. These SnCs have systemic effect through circulating SASP. These systemic inflammatory signals, together with increased local senescence, can effectively prolong or enhance the immune signal that occurs upon injury, pushing it into the fibrotic basin of attraction (Fig S5B). The threshold for fibrosis may thus become lower with age.

In most people and most tissues this threshold is not normally crossed. In people with genetic and environmental conditions that cause increased damage to a given tissue, fibrosis can occur if the damage exceeds the age-related threshold. This can lead to organ-specific fibrotic diseases that are strongly age related, and occur only in a fraction of the population.

Thus, the local, injury-related nature of senescence at young ages versus the systemic nature of SnCs at old ages might contribute to the opposing effects of SnCs on fibrosis.

Transparent Methods

Model for the myofibroblast-macrophage circuit

We model the reciprocal interaction between monocyte-derived-macrophages, *M*, and the ECM-producing myofibroblasts, *mF*. Macrophages secrete PDGF for myofibroblast survival and division, and myofibroblasts secrete CSF for macrophage survival and division (Fig 1C). We assume a mean-field approximation where all cells see the same concentrations of growth factors. The balance of cell proliferation and removal yields equations for the rate of change of cell numbers:

(1) $\dot{mF} = mF\left(\lambda_1 \frac{PDGF}{k_1+PDGF}\left(1-\frac{mF}{K}\right)-\mu_1\right)$

(2) $\dot{M} = M\left(\lambda_2 \frac{CSF}{k_2+CSF}-\mu_2\right)$

where λ_1, μ_1, λ_2, μ_2 are the proliferation (at saturating growth factors) and removal rates of myofibroblasts and macrophages, respectively. The effect of each growth factor on its target cell occurs by binding of the growth factor to its cognate receptor on the target cells, as described by Michaelis–Menten functions with halfway effect at k_1 for PDGF receptor and k_2 for CSF receptor. Myofibroblasts have a carrying capacity, K, defined as the maximum cell population size that can be supported in the tissue, whereas macrophages are assumed to be far from their carrying capacity. Carrying capacity is modelled by a logistic term, as supported experimentally (Zhou et al., 2018).

The equations for the growth factors include production terms, removal by endocytosis and degradation terms, as well as autocrine production of PDGF by myofibroblasts:

(3) $\dot{CSF} = \beta_1 mF - \alpha_1 M \frac{CSF}{k_2+CSF} - \gamma CSF$

(4) $\dot{PDGF} = \beta_2 M + \beta_3\, mF - \alpha_2\, mF \frac{PDGF}{k_1+PDGF} - \gamma PDGF$

Here, CSF is produced by myofibroblasts at rate β_1, and endocytosed by macrophages at maximal rate α_1 (Eq. 3). PDGF is produced by macrophages and myofibroblasts at rates β_2 and β_3 respectively, and endocytosed by myofibroblasts at maximal rate α_2 (Eq. 4). Both growth factors are degraded at rate γ. We assume here that endocytosis works with Michaelis–Menten kinetics with the same halfway points, k_i, as the effect of the growth factors on their target cells in Eqs. 1 and 2, because both signaling and endocytosis depend on ligand binding to the cognate receptor. The reference parameter values are listed in Table 1. Note that Eqs. 3-4 for the growth factors have much faster timescales that Eqs. 1-2 for the cells.

We used the NDSolveValue function in Mathematica 12.0 in order to numerically solve the model (Eqs. 1-4) and plot the phase portraits.

Model for the myofibroblast-macrophage circuit with CSF downregulation by PDGF

As an example of a circuit with two stable fibrosis states, hot and cold fibrosis, we add an interaction to the myofibroblast-macrophage circuit in which PDGF downregulates CSF expression in myofibroblasts. Therefore, the equation for CSF becomes:

(5) $\dot{CSF} = \beta_1 \frac{k_1}{k_1+PDGF} mF - \alpha_1 M \frac{CSF}{k_2+CSF} - \gamma CSF$

Model for ECM accumulation

We modeled the accumulation of ECM (*E*) produced by myofibroblasts. ECM degradation is controlled by proteases, *P*, which are inhibited by anti-proteases *A*. *P* are secreted mainly by macrophages and *A* are secreted by both macrophages and myofibroblasts, resulting in:

(6) $\dot{A} = a\, M + b\, mF - \alpha_A A$
(7) $\dot{P} = P_0 + c\, M - \alpha_P P$

Here, *A* are produced by macrophages and myofibroblasts at rates *a* and *b*, respectively, and degraded at rate α_A (Eq. 6). We assume a basal protease secretion by other cell types, P_0. *P* are produced at rate *c* and degraded at rate α_P. Assuming that *P* and *A* reach their steady-states on a faster timescale than the cells and ECM, we calculate their steady-states by equating Eqs. 6-7 to zero:

(8) $A_{st} = \frac{a}{\alpha_A} M + \frac{b}{\alpha_A} mF$
(9) $P_{st} = \frac{P_0}{\alpha_P} + \frac{c}{\alpha_P} M$

We next consider the equation for ECM, where *P* enhance ECM degradation and *A* inhibit it:

(10) $\dot{E} = \beta_E\, mF - \alpha_E \frac{P_{st}}{A_{st}+k_E} E$

Here, β_E is the production rate of ECM by myofibroblasts and α_E is its removal rate by P. k_E is the halfway point of inhibition of ECM degradation by *A*. Plugging in the *P* and *A* steady states (Eqs 8-9) to Eq. 10 yields:

(11) $\dot{E} = \beta_E\, mF - \alpha_E \frac{\frac{P_0}{\alpha_P}+\frac{c}{\alpha_P}M}{\frac{a}{\alpha_A}M+\frac{b}{\alpha_A}mF+k_E} E$

We define dimensionless variables: $E\frac{\mu_1}{\beta_E K} \to E$, $\frac{A}{k_E} \to A$, $\frac{P}{k_E} \to P$ and dimensionless parameters: $\frac{\alpha_E}{\mu_1} \to \alpha_E$, $a\frac{\gamma k_{21}}{\beta_{21} k_E \alpha_A} \to a$, $b\frac{K}{k_E \alpha_A} \to b$, $c\frac{\gamma k_{21}}{\beta_{21} k_E \alpha_P} \to c$, $\frac{P_0}{k_E \alpha_P} \to P_0$. Eq. 11 now reads:

(12) $\dot{E} = mF - \alpha_E \frac{P_0 + c\, M}{a\, M + b\, mF + 1} E$

Thus, the steady state of ECM is: $E_{st} = \frac{1}{\alpha_E}\frac{mF}{P_0+c\, M}(a\, M + b\, mF + 1)$. The reference parameter values that we used for the ECM dynamics are listed in Table 2.

Simulating inflammatory signals

We model injury scenarios by considering an inflammatory signal, $I(t)$, that represents the influx of monocytes/macrophages. The equation for macrophages thus reads:

(13) $\dot{M} = I(t) + M\left(\lambda_2 \frac{CSF}{k_2+CSF} - \mu_2\right)$

The pulse-like inflammatory signal, of duration τ, is given by:

(14) $I(t) = A_0\big(\theta(t) - \theta(t-\tau)\big)$.

where $\theta(t)$ is the Heaviside step function. We simulated three different signals. For a transient injury, we consider a single pulse with amplitude $A_0 = 10^6 \frac{cells}{day}$ and duration of $\tau = 2$ days. For a repetitive injury, we consider two successive pulses of amplitude $A_0 = 10^6 \frac{cells}{day}$ and duration of $\tau = 2$ days. For a prolonged injury, we consider a pulse with $A_0 = 10^6 \frac{cells}{day}$ and duration of $\tau = 4$ days.

Maturation time of ECM

In order to compute the scar maturation time, we used the FindRoot function in Mathematica 12.0 to numerically find the time when ECM reaches halfway to its final level for different immune pulse durations.

Duration of critical time-window for inflammation

We calculate numerically the duration of the critical time-window for inflammation that results in healing by considering the final level of ECM as function of inflammation pulse duration, $E(\tau)$. This function is similar to a step function moving from a low level to a high level at the time that defines the duration of the time window (Fig 3J). We therefore estimate the time-window duration by computing the derivative of $E(\tau)$, and finding the time in which the derivative is largest. We used the MaximalBy function in Mathematica 12.0 to find the inflammation pulse duration, τ, that maximizes $dE/d\tau$.

Parameters for fibrosis prevention and reversal by eliminating the cold fibrosis fixed point

To analyze the stability of the cold fibrosis fixed point, we consider a situation with zero macrophages. Therefore, the equations that describe myofibroblast dynamics include only myofibroblasts and their autocrine growth factor PDGF. Near the cold fibrosis fixed point, PDGF is mainly removed by endocytosis by myofibroblasts and we therefore assume that we can neglect its non-endocytotic degradation rate γ:

(15) $\dot{mF} = mF\left(\lambda_1 \frac{PDGF}{k_1+PDGF}\left(1-\frac{mF}{K}\right)-\mu_1\right)$

(16) $\dot{PDGF} = \beta_3\, mF - \alpha_2\, mF \frac{PDGF}{k_1+PDGF}$

Using a quasi-steady state approximation due to the faster timescale of growth factors compared to cells, we calculate the PDGF steady state:

(17) $PDGF_{st} = \frac{\beta_3}{\alpha_2-\beta_3} k_1$

Substituting this in the equation for myofibroblasts yields:

(18) $\dot{mF} = mF\left(\lambda_1 \frac{\beta_3}{\alpha_2}\left(1-\frac{mF}{K}\right)-\mu_1\right)$

Solving for the myofibroblast steady state by equating Eq. 18 to zero yields either the healing state ($mF = 0$) or the approximate cold fibrosis fixed point:

(19) $mF_{ON/OFF} \cong \left(1-\frac{\alpha_2\mu_1}{\beta_3\lambda_1}\right)K.$

Eq. 19 provides a condition for the existence of the cold fibrosis fixed point. Since myofibroblast number cannot be negative, the ratio, $C \equiv \frac{\beta_3\lambda_1}{\alpha_2\mu_1}$ must be larger than 1 in order to have a non-zero solution. This means that when C is small enough, the cold fibrosis state will not be a solution and dynamics flow to the healing fixed point.

Supplemental References

Campisi, J. (2005). Senescent cells, tumor suppression, and organismal aging: good citizens, bad neighbors. Cell *120*, 513–522.

van Deursen, J.M. (2014). The role of senescent cells in ageing. Nature *509*, 439–446.

Hecker, L., Logsdon, N.J., Kurundkar, D., Kurundkar, A., Bernard, K., Hock, T., Meldrum, E., Sanders, Y.Y., and Thannickal, V.J. (2014). Reversal of persistent fibrosis in aging by targeting Nox4-Nrf2 redox imbalance. Sci. Transl. Med. *6*, 231ra47–231ra47.

Jun, J.-I., and Lau, L.F. (2010). The matricellular protein CCN1 induces fibroblast senescence and restricts fibrosis in cutaneous wound healing. Nat. Cell Biol. *12*, 676.

Krizhanovsky, V., Yon, M., Dickins, R.A., Hearn, S., Simon, J., Miething, C., Yee, H., Zender, L., and Lowe, S.W. (2008). Senescence of activated stellate cells limits liver fibrosis. Cell *134*, 657–667.

Muñoz-Espín, D., and Serrano, M. (2014). Cellular senescence: from physiology to pathology. Nat. Rev. Mol. Cell Biol. *15*, 482–496.

Piera-Velazquez, S., and Jimenez, S.A. (2015). Role of cellular senescence and NOX4-mediated oxidative stress in systemic sclerosis pathogenesis. Curr. Rheumatol. Rep. *17*, 473.

Rodier, F., and Campisi, J. (2011). Four faces of cellular senescence. J. Cell Biol. *192*, 547–556.

Zhou, X., Franklin, R.A., Adler, M., Jacox, J.B., Bailis, W., Shyer, J.A., Flavell, R.A., Mayo, A., Alon, U., and Medzhitov, R. (2018). Circuit Design Features of a Stable Two-Cell System. Cell *172*, 744-757.e17.
